# A Case for Diagnosis

**Published:** 1905-02-11

**Authors:** 


### A CASE FOR DIAGNOSIS.

Nowhere in medicine is the difficulty of judgment bewailed by Hippocrates more in evidence than in the matter of nervous disease. This is especially the case in women in whom the hysterical counterfeits of organic nervous lesions most frequently obscure the truth, although women have no monopoly in hysteria. The misfortune of this position is emphasised by the fact that in no other two alternative pathological conditions are the prospects of the individual more diametrically opposed or errors in diagnostics more heavily visited. It is hard to say which is the more painful embarrassment, whether to have condemned to a lingering uselessness the patient who suddenly wakes to the conclusion that her palsied extremities are still fit for service, or to have outlined a cheerful future for one whose steady progress to the grave gives a tragic negative to the diagnosis of hysteria.

The difficulties above outlined are well illustrated by an instance recently dealt with by Dr. Ormerod.[1] A woman, aged 36, who had borne three children, and had suffered one miscarriage, for about 12 months had experienced a numbness of her hands and feet with a certain degree of loss of power in them. The only objective abnormality at this time was an exaggeration of the knee-jerk, slight unsteadiness in walking, and a deficiency in the strength of the grasp of the right hand. The paresis gradually increased, with some inco ordination of the upper limbs and some tremor. So far there was nothing about the patient inexplicable on a "functional" hypothesis. But later there appeared the extensor response of the great toe to stimulation of the sole of the foot, the sign which is known as the "Babinski reaction." This was the first evidence of organic disease of the nervous system, and it is a sign the diagnostic import of which cannot be over-estimated. There appeared also ankle-clonus, and some loss of pain-sense in the right lower limb. The electrical reactions were normal, as were the sphincters, and there was no nystagmus.

Dr. Ormerod summarises the conditions which, apart from Babinski's sign, inclined him towards the diagnosis of organic disease, and since they are of wide application in cases of this class they will bear recapitulation. Firstly, there was persistent numbness of all extremities, which is uncommon in hysteria, at all events apart from anæsthesia. Secondly, the uselessness of the limbs, though not extreme, was progressive. Thirdly, there were in this patient none of the tender points so common in hysterical women.

Assuming an organic lesion, the diagnosis lay between :—

1. Peripheral neuritis.
2. Disseminated sclerosis.
3. Sub-acute combined degeneration of the spinal cord.

Peripheral neuritis was dismissed on the grounds that there was no pain, tenderness, muscular wasting or alteration in electrical excitability in the affected limbs. Also the reflexes pointed to a lesion of the upper segment.

Disseminated sclerosis could not be dismissed but was rendered unlikely by the absence of sudden fluctuation in the symptoms, and by the absence of nystagmus, intention tremor and sphincter trouble.

The author inclined to the third alternative, a little known disease, but most important on account of its rapid fatality. The symptomatology falls into three stages—(1) A period of subjective sensations in the limbs—numbness, tingling, pins-and-needles—with mild paralytic phenomena of a spastic type, *i.e.*, with increased reflexes and an extensor plantar response; (2) a period of increasing spastic paralysis and of anæsthesia spreading from below upward ; (3) a period when the paralysis has become flaccid, the knee-jerks disappear though the plantar reflex remains extensor, the sphincters become involved, and the patient dies in a state of generalised paralysis, the duration of the illness being from one to two years.

[1] Clin. Jour., Jan. 1905.

